# Predicting Psychotic-Like Experiences during Sensory Deprivation

**DOI:** 10.1155/2015/439379

**Published:** 2015-02-24

**Authors:** Christina Daniel, Oliver J. Mason

**Affiliations:** Research Department of Clinical, Educational and Health Psychology, University College London, London WC1E 7HB, UK

## Abstract

*Aims*. This study aimed to establish the contribution of hallucination proneness, anxiety, suggestibility, and fantasy proneness to psychotic-like experiences (PLEs) reported during brief sensory deprivation. *Method*. Twenty-four high and 22 low hallucination-prone participants reported on PLEs occurring during brief sensory deprivation and at baseline. State/trait anxiety, suggestibility, and fantasy proneness were also measured. *Results*. Both groups experienced a significant increase in PLEs in sensory deprivation. The high hallucination prone group reported more PLEs both at baseline and in sensory deprivation. They also scored significantly higher on measures of state/trait anxiety, suggestibility, and fantasy proneness, though these did not explain the effects of group or condition. Regression analysis found hallucination proneness to be the best predictor of the increase in PLEs, with state anxiety also being a significant predictor. Fantasy proneness and suggestibility were not significant predictors. *Conclusion*. This study suggests the increase in PLEs reported during sensory deprivation reflects a genuine aberration in perceptual experience, as opposed to increased tendency to make false reports due to suggestibility of fantasy proneness. The study provides further support for the use of sensory deprivation as a safe and effective nonpharmacological model of psychosis.

## 1. Introduction

Although most commonly associated with psychiatric disorders, it is now acknowledged that during their lifetime, about 28% of the general population may have psychotic-like experiences (PLEs), at least as detected by screening questions in the US National Comorbidity Survey [1]; for review, see [2]. These may include hallucinations, passivity phenomena, and overvalued or delusional ideas. Other experiences phenomenologically more distal to psychosis, such as belief in having had “psychic” or paranormal experiences (e.g., telepathy, ESP, telekinesis, and “out-of-body” experiences), synaesthesia, lucid dreaming, and hypnopompic/hypnagogic states, occur even more widely in the population. Having been termed “anomalous experiences,” such experiences have variously been associated with schizotypy (a latent personality construct representing psychosis-proneness [3]) and thus may form part of the broader constellation of quasipsychotic phenomena.

There is a long history of experimental paradigms attempting to induce anomalous experiences in healthy individuals, much of it taking place back in the 1950s and 1960s. Many of these studies employed sensory deprivation of various kinds though findings were inconsistent, possibly due to an inadequate recognition of the complexity of variables relevant to sensory restriction [4]. Prolonged periods of deprivation were found to produce a range of quasipsychotic phenomena in many, if not all, participants. However, experiences at shorter durations varied depending on the nature of the deprivation and the characteristics of the participants involved. Other studies [5–7] concluded that anomalous experiences occur in highly suggestible individuals who have a tendency to mistake “imaginary” events as being “real.” Researchers lost interest in the field of inducing anomalous experiences, many dismissing the phenomena as more akin to fantasy or acts of imagination and not a true parallel of the hallucinations and other positive symptoms seen in psychosis. In recent years, interest has returned to anomalous experiences within the normal population, whether conceived as part of a psychosis continuum [2] or the theoretical construct of schizotypy. This is a set of subclinical characteristics thought to indicate a raised risk of psychotic illness that in some ways are milder versions of clinical signs and symptoms. There are now a number of questionnaires to measure schizotypy: for example, the Oxford-Liverpool Inventory of Feelings and Experiences (O-LIFE-B, [8]) and the Launay-Slade Hallucination Scale (LSHS, [9]), as well as measures of PLEs such as the Community Assessment of Psychic Experiences (CAPE, [10]). Furthermore, the study of individuals with schizotypal characteristics such as hallucination-proneness has the advantage that results are not confounded by the contribution of variables such as hospitalization, medication effects, illness duration, and cognitive deficits.

With the potential utility for studying PLEs in the normal population now clearly reestablished, researchers have taken a fresh look at methods to experimentally induce such experiences and have employed a variety of different techniques including sensory deprivation [11–17], ambiguous auditory paradigms [18–22], perceptually ambiguous visual paradigms [23–25], mirror gazing [26–29] and naturalistic experiments [30]. For a detailed review of these methods, see Daniel and Mason [31]. Many of these methods have been informed by recent theoretical accounts of “voice hearing” such as increased impact of top-down processing [32, 33]; reality discrimination failure [34]; and increased sensitivity to internally-generated percepts [35]. Overall, several studies suggest a tendency to erroneously allocate an external source to internally generated stimuli may underlie hallucination proneness in schizophrenia (for review see [36]). By extension, these findings support a tentative hypothesis that in sensory deprivation, where external events are absent or minimal, highly schizotypal individuals are more likely to erroneously process inner thoughts as being external events leading to hallucinatory phenomena.

Seven studies attempting to use a sensory deprivation paradigm to induce quasipsychotic experiences in the normal population have been published since 1990 [11–17]. Using more modern techniques, all studies reported anomalous perceptions of varying complexity in many of the participants. Furthermore, PLEs have been shown to be successfully induced using such methods in as little as 15 minutes' exposure [14]. The impetus for the current study comes from a small pilot study [14] that investigated the potential of using an anechoic chamber (an environment of near-total light and sound deprivation) to induce PLEs in healthy participants scoring either high or low for hallucination proneness. PLEs taking the form of perceptual disturbances, paranoia, and anhedonia were found in sensory deprivation. However, there were a number of methodological limitations. Recruitment was on a relatively small scale (*n* = 19), and the study is therefore likely to have been underpowered. The study has also been criticised by Bell [37] for including a panic button, on the basis that a previous study [38] exploring the impact of a panic button showed the group with the button reported many more perceptual aberrations and cognitive and emotional disturbances, including heightened anxiety. Bell [37] also suggested that greater PLEs in the high hallucination prone group might be accounted for by differential anxiety levels between high and low-prone groups (not measured) since hallucination proneness has been linked to trait anxiety [39].

Another issue relating to the induction of PLEs in normal populations is whether some individuals have a greater tendency to merely endorse items, even if they have not actually experienced them. Merckelbach and van de Ven [20] used a white noise paradigm to investigate whether reports of hallucinatory experiences were associated with a heightened sensitivity to demand characteristics, suggestibility, and fantasy proneness. Results showed that reports of hallucinations were actually best predicted by fantasy proneness rather than hallucinatory disposition, calling into question the validity of conclusions drawn from previous research that show a significant proportion of the normal population may have hallucinatory experiences. They suggest that hallucination proneness in the normal population is closely associated with fantasy proneness, and it is fantasy proneness that leads participants to endorse odd experiences (even if they have not actually experienced them). However, an alternative interpretation of Merckelbach and van de Ven's [20] findings is that fantasy proneness mediates the process by which highly prone individuals experience hallucinations. In this vein, Bentall [34] has suggested that fantasy proneness drives a specific response bias reflecting impaired reality testing, which in turn leads to reports of hallucinations. Clearly, this issue requires further study, as fantasy proneness has not routinely been measured in nonclinical studies of hallucinations.

Evidence in support of the normal population experiencing true hallucinatory experiences during ambiguous auditory paradigms (as opposed to merely endorsing them) comes from experimental studies that have examined similarities between clinical groups with a diagnosis of psychosis and high hallucination prone individuals from within the normal population. In accordance with a continuum model of psychosis [40] the rate that individuals from the normal population report hearing hallucinations in random noise has been shown to be progressively greater across groups with increasing familial risk for psychosis [21]. Individuals from the normal population reporting hallucinations during ambiguous auditory tasks have also been shown to mirror clinical populations in terms of psychosis risk factors, including being younger in age and more likely to be male than female [22]. Whilst there is mounting evidence to suggest that experimentally induced hallucinations are not purely the product of demand characteristics and fantasy proneness, a number of studies that have measured these variables report they are associated with increased likelihood of anomalous experiences. As these studies are correlational in design, it is not possible to implicate these factors in the causality of hallucinations. In particular, it remains unclear whether fantasy-proneness is associated with increased reports of hallucinations through direct causality, or whether this trait is associated with high schizotypy, and it is schizotypal tendencies that drive the experience of hallucinations. Studies that have directly compared high and low schizotypal groups have not generally incorporated measures of fantasy proneness, and there is a need for future studies to do so.

*Aims and Hypotheses.* The current study aimed to establish, with greater clarity, the effects of brief sensory deprivation (using an anechoic chamber) on individuals who vary in their degree of hallucination proneness (schizotypy). The initial design of Mason and Brady's study [14] was modified in order to address some of the methodological limitations discussed above and also to answer some additional research questions. Key modifications included measuring state and trait anxiety before and during the experiment and incorporating additional measures into the design, including suggestibility and fantasy proneness. A one-way microphone was also used to monitor participants rather than using a panic-button in an attempt to reduce potential demand characteristics.

The presence of PLEs was evaluated under normal baseline conditions and in sensory deprivation conditions produced by using an anechoic chamber. A group of participants who were rated highly for schizotypy was compared against a group who rated low for such traits. It was hypothesised that

1. sensory deprivation would be associated with greater PLEs when compared to baseline after controlling for anxiety, suggestibility, and fantasy proneness;
2. the high hallucination prone group would report greater PLEs than the low prone group when under sensory deprivation, after controlling for anxiety, suggestibility, and fantasy proneness;
3. both hallucination proneness (high/Low group membership) and fantasy proneness will predict the increase in PLEs reported in sensory deprivation.

## 2. Method

### 2.1. Participants

Participants between the ages of 18 and 65 years were recruited via a university-wide email sent out to all students and staff. Exclusion criteria included a history of a major psychiatric or neurological disorder or current recreational drug use (defined as during the last three months). The email invited participants to complete a 126 item online questionnaire, comprising a brief fantasy proneness measure (The Creative Experiences Questionnaire, [41]); The Marlow-Crowne Social Desirability Scale Short-form (MC-13) [42]; a brief measure of hallucination proneness (The Revised Hallucinations Scale (RHS), [43]).

Five hundred sixty-two participants from a wide range of ethnic backgrounds returned completed questionnaires. Initially, only the RHS scores were examined, and the highest 10% of scorers and lowest 20% of scorers were identified from the sample. The top decile is frequently chosen as it may represent a “taxon” group that possesses a true risk of developing future psychosis [44]. A rather wider low hallucination prone group was chosen so as not to contain only individuals with extreme scores of this kind who may perhaps represent an unsuitable reference group. This resulted in the high RHS group containing individuals with scores ≥52 (*n* = 60) and the low group containing individuals with scores ≤31 (*n* = 131). Both groups were then formed into randomised lists using an online randomisation tool (https://www.random.org/), and participants were invited to take part in list-wise order. Thirty-five participants in the high scoring group and 29 in the low scoring group were invited to participate in the experiment. Of these, 24 high scorers (13 males, 11 females, mean age = 21.25 years, SD = 3.38, mean score = 58.17, and SD = 6.51) and 22 low scorers (7 males, 15 females, mean age = 28.23 years, SD = 9.10, mean score = 27.77, and SD = 1.82) attended and took part. Informed consent was obtained, and the study was ethically approved according to university regulations.

### 2.2. Power Analysis

Very little is known about the effects of sensory deprivation on people who rate highly for hallucination proneness, and so it was challenging to accurately estimate effect sizes from existing literature. The most similar study to date [14] reported large effect sizes for increases in perceptual distortions (partial eta squared = 0.56) and anhedonia (partial eta squared = 0.58) measured using the Psychotomimetic States Inventory [45] immediately after 15 minutes of sensory deprivation. The power calculation for the current study was based on the smallest of these effect sizes reported by Mason and Brady [14]: partial eta squared = 0.56. This is a conservative estimate for current purposes since participants in the current study spent a longer length of time in sensory deprivation (25 minutes) presumably providing greater opportunity for perceptual distortions to arise. Power calculations suggested that a minimum total sample of *N* = 36 (i.e., 18 per group) would provide statistical power for a between-within participants repeated measures ANOVA design that exceeded 80% (*β* = 0.80), with *α* = 0.05.

### 2.3. Measures

#### 2.3.1. Gudjonsson Suggestibility Scale (Short Version) (GSS) [46]

The test consists of a short narrative read to the person, immediately followed by twenty questions about what they have heard. Fifteen questions are loaded with suggestion, whereas five are not. The person is requested to answer the questions as accurately as they can. “Yield” suggestibility is a measure of how much participants give in or yield to the 15 suggestive questions. After giving their answers they are then told that there are errors in their answers and must answer the questions a second time. “Shift” suggestibility is a measure of how much participants' responses can be shifted by pressured instructions. Several studies have supported the scales' criterion-related validity [46–48]. As the current study was concerned with the impact of potentially suggestive questions contained in questionnaires surveying perceptual distortions, “Yield” suggestibility was used as the measure best reflecting this tendency.

#### 2.3.2. The Creative Experiences Questionnaire (CES) [41]

A self-report measure of fantasy proneness consisting of 25 yes/no items. The scale has been shown to have good test-retest stability (*r* = 0.95) and adequate internal consistency over a six week period (Chronbach's alpha = 0.72). The scale has also been shown to have good construct validity against an earlier measure of fantasy proneness (the Inventory of Childhood Memories and Imaginings, [49, 50]).

#### 2.3.3. The Revised Hallucinations Scale (RHS) [43]

This is a 24-item questionnaire based on the Launay-Slade Hallucination Scale [9] measuring a predisposition to experience hallucinations. It uses a revised scoring method which allows participants to respond on a 4-point scale (1 = never to 4 = almost always). The scale has been shown to have good reliability and predictive validity and moderately stable internal consistency over a period of 4–6 weeks [29].

#### 2.3.4. The Psychotomimetic States Inventory (PSI) [45]

This is a 48 item questionnaire measuring psychosis-like experiences. Items are rated on a 4-point scale (from 0 = never to 3 = strongly), with some items being reverse scored. The Psychotomimetic States Inventory has subscales of Delusory Thinking, Perceptual Distortions, Cognitive Disorganization, Anhedonia, Mania, and Paranoia. It was originally developed for use in drug studies, and it was used here because there are currently no validated measures available specifically for studying the effects of sensory deprivation. Despite the limitations of using a non-validated measure, the PSI has produced meaningful results in a previous preliminary study of sensory deprivation [14], and therefore it was included in the current study to further validate the measure in this context.

#### 2.3.5. The State-Trait Anxiety Inventory (STAI) [50]

A pair of two 20-item questionnaires measure the temporary condition of state anxiety and the more longstanding quality of trait anxiety. Items are rated on a 4-point scale. The STAI has been shown to have good construct validity with multiple other assessment tools [51]. It has also been shown to have good test-retest reliability (0.54) correlation for state, and 0.86 correlation for trait anxiety [52].

### 2.4. Equipment

An anechoic chamber was used to produce the deprivation condition. The anechoic chamber is constructed as a room within a room (see http://www.langsci.ucl.ac.uk/resource/anechoicroom.html). The outer walls are 330 mm thick and the inner room is formed of metallic acoustic panels mounted on a floating floor which is then lined with large glass fibre wedges. This results in a very low noise environment in which the sound pressure due to outside levels is below the threshold of human hearing. It is also possible to remove all sources of light from the room and thus create an environment with near complete deprivation of sight and sound.

### 2.5. Procedure

On arrival at the testing facility, participants were given a short briefing by the experimenter, in a calm and reassuring tone. Participants were informed that they would “experience what it is like to spend a short period of time (less than half an hour) in sensory deprivation” and that this would “involve being alone in a room with zero light and sound.” Due to the ethical need to inform participants about any potential negative aspects of taking part, they were also briefed that “since people do not normally experience sensory deprivation in their day-to-day lives, there was a small risk they would find the experience stressful or that they may have some unusual sensory experiences.” No other information was given about the research hypotheses in order to avoid influencing participants' responses.

Participants initially completed the trait anxiety measure of the STAI. Participants were then given a demonstration of the anechoic chamber so that they could familiarise themselves with the environment. They were then asked to sit in silence in the anechoic chamber in a padded armchair in the middle of the room. Participants were informed that they would be spending approximately 25 minutes in the chamber in complete silence and darkness. It was explained that a microphone was present in the chamber so that participants could be heard by the experimenter outside should they become distressed. This was a one-way set-up, and they could not converse with the experimenter. Participants were informed that if they wished to terminate the experiment at any point they should remain seated and tell the experimenter, who would immediately restore light and communication. No participants chose to terminate the experiment early. After completion of 25 minutes within the chamber, participants were moved to an ante-room where they were immediately asked to complete questionnaires referring to the time that they had spent in the anechoic chamber: The State-Trait Anxiety Inventory (state items only); The Psychotomimetic States Inventory. Participants then listened to the narrative and associated questions comprising the Gudjonsson Suggestibility Scale. This took approximately 20 minutes and also acted as a distraction task to allow any effects of the sensory deprivation to dissipate. Participants finally completed a second version of the Psychotomimetic States Inventory, referring to their current baseline state, and the State-Trait Anxiety Inventory (state items only) referring to how they were feeling at that moment in time. Following completion of the experiment, participants were debriefed and received a nominal fee for their time in taking part.

## 3. Results 

### 3.1. Overview of Statistical Treatment

All statistical analyses were conducted using SPSS 22.0. Data were checked for normality before analysis using descriptive statistics and histograms with normal distribution curves. All self-report scores were normally distributed, meeting parametric assumptions. A marked difference in gender distribution between the two groups was noted (with the high scoring group (*n* = 24) consisting of 13 males and 11 females, and the low scoring group (*n* = 22) consisting of 7 males, 15 females). Following baseline comparisons of the groups using MANOVA, a repeated measures ANCOVA was conducted to test hypotheses relating to changes in psychotic-like experiences across conditions taking covariates into account. Gender was included as a covariate in order to control for the effect an uneven gender distribution may otherwise have had on between-group results. Unfortunately, trait anxiety had to be excluded as a covariate in this analysis due to a significant interaction effect, violating the assumption of homogeneity of regression slopes. Subsequently, in order to test the hypothesis that both hallucination proneness and fantasy proneness predict the increase in PLEs reported in sensory deprivation, a stepwise regression was run to determine the impact of Group and the additional covariates on PSI scores in sensory deprivation. Finally, state anxiety was investigated using a repeated measures ANOVA.

### 3.2. Baseline Group Comparisons

It was hypothesised that the high hallucination prone group would score significantly more highly on the PSI under normal baseline conditions. MANOVA showed the high and low schizotypy groups differed on all baseline measures, with the high scoring group reporting a greater number of psychotic-like experiences consistent with the first hypothesis (see Table 1 for descriptives).

Although relationships with anxiety, suggestibility, and fantasy proneness were not hypothesised, all the above findings are in the expected direction (see Table 2). Baseline suggestibility was measured using the yield subscore of the Gudjonsson Suggestibility Scale as this specifically focuses on the impact of suggestive questions, the type of suggestibility likely to have had most potential impact on participant responses to questionnaires during this study. A significant difference in suggestibility was found between the high and low schizotypy groups (*F*(1,45) = 9.18, *P* < 0.01), with the high schizotypy group being more suggestible (mean = 4.46, SD = 2.83) than the low schizotypy group (mean = 2.18, SD = 2.20). Further analysis revealed that the differences in suggestibility between groups could not be attributed to differences in the memory recall component of the suggestibility task (*F*(1,45) = 0.42, *P* > 0.05), indicative of a true difference in suggestibility between the groups as opposed to reflecting differing recall ability.

Due to the large number of baseline variables that differed significantly between the two groups, correlations were calculated between all baseline variables measured and the dependent variable of interest (PSI) to assess for their relevance as covariates in ANCOVA, using Bonferroni adjusted alpha levels of  0.003 per test (0.05/15). Trait anxiety, baseline state anxiety, deprivation state anxiety, and fantasy proneness were all found to be significantly positively correlated with baseline psychosis-like experience scores. Baseline state anxiety, deprivation state anxiety, and fantasy proneness were also found to be significantly positively correlated with sensory deprivation psychosis-like experience scores. Suggestibility was not found to be significantly correlated with psychosis-like experience scores in either condition. Consequently, baseline state anxiety, deprivation state anxiety, and fantasy proneness were considered as covariates for analysis of variance for PSI scores (plus gender as discussed above). As mentioned previously, trait anxiety could not be included in the ANCOVA due to a violation of statistical assumptions.

### 3.3. ANCOVA: PLEs across Groups and Conditions

It was hypothesised that there would be a significant increase in psychotic-like experiences from baseline in sensory deprivation across both groups. A mixed between-within subjects repeated measures analysis of variance was run, with baseline state anxiety, deprivation state anxiety, fantasy proneness, and gender controlled for as covariates. Results demonstrated a significant main effect of condition for PSI scores, *F*(1,40) = 7.09, (*P* = 0.01) (see Table 3 for descriptives). This indicates that, overall, participants experienced a significantly greater number of psychosis-like symptoms during sensory deprivation than at baseline.

**Figure 1:**
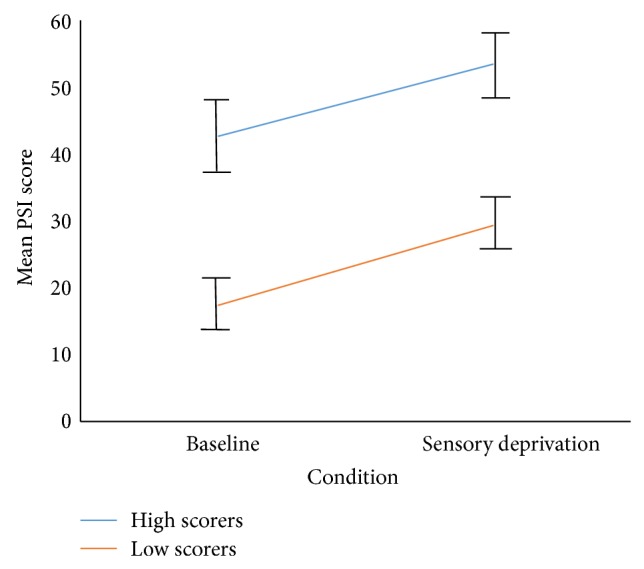
Mean PSI scores in high and low schizotypy groups by condition.

The mixed ANCOVA analysis did not show a significant main effect of group for PSI scores, *F*(1,40) = 3.73, (*P* = 0.06) (see Table 3 for descriptives). This indicates that the high and low hallucination prone groups reported similar levels of psychosis-like symptoms overall throughout the experiment. However, Figure 1, showing mean PSI scores in the high and low schizotypy groups by condition (unadjusted for covariates), together with the PSI scores in Table 3, depicts the high scoring group having markedly higher PSI scores throughout the experiment. It is therefore likely that adjustment for the relatively large difference in gender distribution between the two groups is responsible for the lack of a significant main effect of group in the analysis.

Further mixed between-within subjects repeated measures ANCOVAs examining the PSI subscales of Delusional Thinking, Perceptual Distortion, Cognitive Disorganisation, Anhedonia, Mania, and Paranoia were conducted, once again controlling for baseline state anxiety, deprivation state anxiety, fantasy proneness, and gender as covariates, to investigate any difference in particular types of PLEs reported. Analysis revealed a significant main effect of condition for Perceptual Distortions, *F*(1,40) = 9.19, *P* = 0.00. Perceptual distortions were significantly higher in sensory deprivation than at baseline. A significant main effect of group was found for the Anhedonia subscale (*F*(1,40) = 5.46, *P* = 0.03), with high scorers showing greater levels of anhedonia throughout the experiment overall. No significant main effects were found for the subscales of Delusional Thinking, Cognitive Disorganisation, or Paranoia (see Table 4).

### 3.4. Regression: Impact of Group and Covariates on Sensory Deprivation PSI Scores

A post hoc stepwise regression analysis was run to determine the impact of Group and the individual covariates on PSI scores in sensory deprivation. This yielded a final two-factor model (containing the factors Group and Deprivation State Anxiety) that was able to account for 54% of the variance in deprivation PSI scores (*F*(2,43) = 24.75, *P* < 0.001, *R* ^2^ = 0.54), significantly more than that utilising group alone (See Table 5 for full details of both models). The final regression model showed state anxiety levels were a significant predictor of psychosis-like experiences in sensory deprivation. However, group proved to be a more powerful predictor of psychosis-like experiences in sensory deprivation (accounting for 39% of the variance, compared to 15% for the unique contribution of state anxiety alone).

### 3.5. ANOVA: State Anxiety across Groups and Conditions

**Figure 2:**
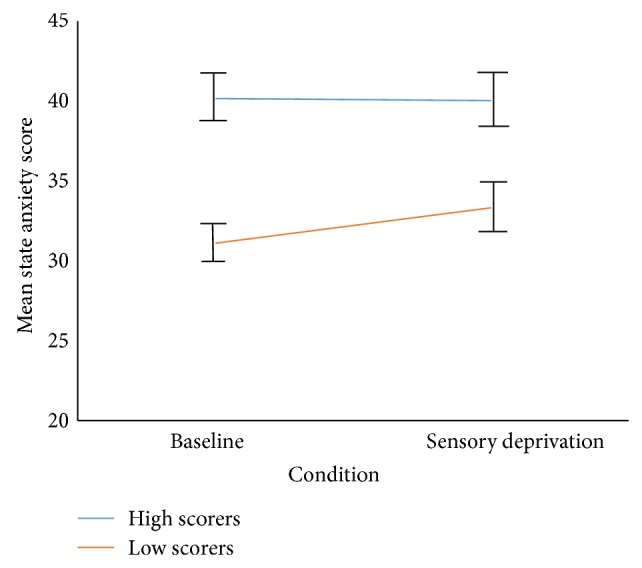
State anxiety scores in high and low schizotypy groups by condition.

To test the potential role that changes in anxiety might play in sensory deprivation, a mixed between-within subjects repeated measures ANOVA was conducted for state anxiety. This demonstrated a significant main effect of group for state anxiety scores, *F*(1,44) = 7.98, (*P* < 0.01) (see Table 3 for descriptives): the high hallucination prone group experienced greater state anxiety than the low hallucination prone group. There was no effect of condition, suggesting that state anxiety did not differ between baseline and sensory deprivation conditions (see Figure 2). Therefore, the increased psychosis-like symptoms experienced by both groups in sensory deprivation cannot readily be attributed to increased state anxiety levels.

## 4. Discussion

Consistent with the hypotheses, both high and low hallucination prone groups experienced a significant increase in psychosis-like symptoms from baseline in the sensory deprivation environment, and these remained after controlling for state anxiety, suggestibility, and fantasy proneness. As predicted there were marked group differences: the high hallucination prone group reported more psychosis-like experiences at both baseline and in sensory deprivation. These findings are consistent with previous research [14] that, until now, has not taken these potential confounds into account. This provides more substantive evidence that the increase in psychosis-like experiences found in sensory deprivation reflects a genuine aberration in perceptual experience, as opposed to an increased tendency to make reports of psychosis-like experiences driven by individual differences in certain personality traits.

The two groups did, however, exhibit differences on a number of measures at baseline, with the high scoring group reporting greater state/trait anxiety, greater suggestibility, and greater fantasy proneness. All of these findings are broadly consistent with existing literature showing anxiety to be linked to hallucination proneness [39] and acute anxiety in individuals with clinical psychosis linked to an increase in hallucinatory experiences [53]. Fantasy proneness has also previously been shown to be high in individuals who make hallucinatory reports during auditory experimental paradigms [20]. However, results of the stepwise regression showed that, of the between-group differences found in this study, only state anxiety made a significant contribution to change in PSI scores in sensory deprivation. Fantasy proneness and suggestibility were not found to play a significant role. Taken together, these findings may provide an answer to a question posed in the literature that has previously gone unanswered: Is fantasy proneness responsible for a wide variety of atypical reports (including hallucinatory reports) that are unrelated to genuine experiences or does this trait reflect impaired reality testing that gives rise to odd and schizophrenia-like experiences? [20]. The findings of this study support the argument that fantasy proneness (and suggestibility for that matter) are not responsible for hallucinatory reports, and the increased fantasy proneness and suggestibility seen in the high scoring group are likely to reflect other aspects of underlying differences in schizotypal traits (potentially such as impaired reality testing, although this hypothesis remains untested in the current study).

The regression model showed state anxiety levels were a significant predictor of psychosis-like experiences in sensory deprivation. However, Group membership proved to be a more powerful predictor of psychosis-like experiences in sensory deprivation (accounting for 39% of the variance, compared to 15% for the unique contribution of state anxiety alone). This finding was corroborated by ANCOVA showing that the main effect of condition remained once state anxiety had been controlled for as a covariate. Although the high hallucination prone group had higher state anxiety scores, state anxiety remained stable across conditions for both groups. Therefore, although state anxiety was shown to be a predictor of psychosis-like experiences in sensory deprivation, it seems unlikely that anxiety is solely responsible for PLE's, indeed it may be a consequence of PLEs as this study was not designed to test the direction of this relationship.

Sensory deprivation was found to produce a significantly greater increase in the hallucination-prone group on the Perceptual Distortions subscale of the Psychotomimetic States Inventory after controlling for anxiety, suggestibility, and fantasy proneness. This finding was highly marked and consistent with previous studies [14, 17]. However, unlike previous studies, no significant state/trait interactions were found on other PSI scales. These differing findings may be due to methodological differences between the studies, such as length of time in deprivation and statistical control for suggestibility and fantasy proneness.

### 4.1. Strengths and Limitations

Despite attempting to address several potential confounds of previous research, there is some way to go before concluding the phenomena seen in sensory deprivation are equivalent to clinical and nonclinical psychotic experiences. The current study is limited by reliance on self-report measures. Biometric approaches such as psychophysiological or neurocognitive indices would clearly strengthen the argument. However, the inclusion of additional measures of state anxiety, suggestibility, and fantasy proneness is a strength of the study, enabling us to conclude that whilst state anxiety does appear to play a role in the genesis of PLE's, fantasy proneness, and suggestibility are not implicated. The group design (replicating previous studies) meant that hallucination proneness was not used as a continuous variable and it is possible that the study is better powered to detect a difference in this as opposed to the continuously measured variables. However, without a much larger sample it is not possible to correct for this. In addition, trait anxiety could not be included in the analysis, and therefore it is not currently possible to draw further conclusions regarding the potential role trait anxiety may have in the genesis of PLE's (though we would note that trait and state anxiety correlated very highly indeed). Whilst attempts were made to control for the differences in gender distribution between the two groups, this made the results more complex to interpret. The greater proportion of males present in the high scoring group (13 males, 11 females), versus the low scoring group (7 males, 15 females) is perhaps not unexpected given that participants were selected for hallucination proneness, a trait synonomous with schizotypy and psychosis-proneness: it has been widely documented that psychosis is more common in males and with a younger age of onset (for a review see [54]).

Recruitment took place on a university campus, and whilst every effort was made to include staff as well as students to sample a broad age range, the majority of participants were students. This limits the ecological validity of the study, although the impact it is likely to have had on the data is uncertain. Schizotypy scores (and hence hallucination proneness) show a tendency to reduce with increasing age [55], and hence an uneven age distribution between groups (as well as gender) could become problematic in a future study sampling the general population. However, other factors such as education level and IQ would be more representative in a general population as opposed to student sample.

There is now mounting evidence to suggest that using short-term sensory deprivation as a method to induce PLE's in healthy participants is both effective and safe, with over 100 participants now tested across 3 separate studies (see [14, 17]). No adverse long-term effects have been reported, and although the sensory deprivation environment is sometimes experienced as unpleasant, it is generally well tolerated by participants, and there have not been any occurrences of participants requesting to withdraw from any studies to date. In comparison to the risks inherent in using pharmacological paradigms, sensory deprivation would seem a worthy focus for further research.

## 5. Conclusions

Overall the study provides further support for use of sensory deprivation as a nonpharmacological tool for temporarily inducing psychosis-like experiences. Both high and low hallucination prone groups responded to sensory deprivation in a qualitatively similar manner, but with quantitative differences in the frequency of psychosis-like experiences reported, corroborating previous findings. Furthermore, this study provides initial evidence in support of increase in psychosis-like experiences reflecting a genuine aberration in perceptual experience, as opposed to an increased tendency to make reports driven by individual differences in certain personality traits. Increased anxiety, fantasy proneness, and suggestibility were characteristics of the high scoring group, but only anxiety was found to be a predictor of psychosis-like experiences in sensory deprivation. However, group differences in hallucination proneness proved to be the most powerful predictor of psychosis-like experiences.

## Figures and Tables

**Table 1:** Mean scores (SDs) for high and low RHS groups at baseline.

	High (*n* = 24)	Low (*n* = 22 )	*F*	Sig.
RHS	58.17 (1.33)	27.77 (1.82)	446.38	<0.001
PSI	43.04 (17.20)	17.55 (11.20)	34.80	<0.001
Trait anxiety (STAI)	45.92 (12.13)	36.36 (8.78)	9.22	<0.01
Baseline state anxiety	40.21 (11.42)	31.14 (8.62)	9.12	<0.01
MISS	58.46 (18.99)	40.55 (7.39)	17.17	<0.001
GSS yield	4.46 (2.83)	2.18 (2.20)	9.18	<0.01
CES	14.08 (3.89)	4.95 (2.66)	84.72	<0.001

**Table 2:** Correlations between baseline measures, anxiety measures, and PSI scores.

	Trait STAI	Baseline state STAI	Deprivation state STAI	GSS	CES
Baseline PSI	0.74^*^	0.67^*^	0.51^*^	0.30	0.67^*^
Sensory deprivation PSI	0.42	0.52^*^	0.55^*^	0.30	0.51^*^

^*^Correlation is significant at the Bonferroni adjusted alpha level of 0.003 (2-tailed).

**Table 3:** Mean anxiety and PSI scores for high and low schizotypy groups by condition.

	High scorers (*n* = 24)		Low scorers (*n* = 22)	
	Baseline	Deprivation	Baseline	Deprivation
State STAI	40.21 (11.42)	40.08 (12.06)	31.14 (8.62)	33.36 (10.08)
PSI	43.04 (17.20)	53.92 (17.88)	17.55 (11.20)	29.59 (12.71)

**Table 4:** Mean PSI subscale scores for high and low RHS groups by condition.

PSI	High RHS (*n* = 18)		Low RHS (*n* = 18)	
	Baseline	Deprivation	Baseline	Deprivation
Delusory thinking	4.58 (2.59)	6.00 (3.24)	2.50 (2.39)	1.95 (2.56)
Perceptual distortions	3.75 (3.37)	12.29 (6.00)	1.14 (1.73)	7.00 (4.47)
Cognitive disorganisation	13.83 (6.60)	13.71 (6.25)	5.32 (4.26)	6.73 (4.46)
Anhedonia	8.25 (4.63)	9.88 (4.24)	3.14 (3.11)	8.18 (4.23)
Mania	7.21 (2.27)	7.83 (3.07)	4.18 (2.24)	4.32 (2.26)
Paranoia	5.42 (4.10)	4.21 (3.36)	1.27 (1.61)	1.41 (2.02)

**Table 5:** Stepwise regression predicting deprivation PSI scores.

Deprivation PSI scores				
	Model 1		Model 2	
Variable	*B*	*β*	*B*	*β*
Constant	5.27		−13.06^**^	
Group	24.32^**^	0.62	19.70^**^	0.50
Deprivation state anxiety	Excluded		.69^**^	0.40
Fantasy proneness	Excluded		Excluded	
Gender	Excluded		Excluded	
*F*	27.82^**^		24.75^**^	
Δ*R* ^2^	.39		.15	
Δ*F*	27.82^**^		13.67^**^	

Note: *N* = 46. ^**^ *P* < 0.001.
